# Nutritional Status of Children with Short Stature Is Oppositely Associated with Growth Hormone Peak in Stimulation Tests and Insulin-like Growth Factor-1 Concentration

**DOI:** 10.3390/jcm15093333

**Published:** 2026-04-27

**Authors:** Joanna Smyczyńska, Urszula Smyczyńska, Maciej Hilczer, Renata Stawerska

**Affiliations:** 1 Department of Pediatrics, Diabetology, Endocrinology and Nephrology, Medical University of Lodz, 90-419 Lodz, Poland; 2 Department of Biostatistics and Translational Medicine, Medical University of Lodz, 92-215 Lodz, Poland; urszula.smyczynska@umed.lodz.pl; 3 Department of Endocrinology and Metabolic Diseases, Polish Mother’s Memorial Hospital—Research Institute, 93-338 Lodz, Poland; maciej.hilczer@umed.lodz.pl (M.H.); renata.stawerska@umed.lodz.pl (R.S.); 4 Department of Developmental Age and Adult Endocrinology, Medical University of Lodz, 93-338 Lodz, Poland

**Keywords:** short stature, growth hormone deficiency, insulin-like growth factor-1, nutritional status, machine learning, prediction models

## Abstract

**Background/Objectives**: A blunted growth hormone (GH) response in stimulation tests (GHSTs) in obese patients is well documented, with less evidence for insulin-like growth factor-1 (IGF-1) concentrations. The aim of this study was to assess the relationships between nutritional status, GH peak in GHST, and IGF-1 concentrations, and to develop machine learning prediction models of GH deficiency (GHD) in children with short stature. **Methods**: A case–control study included 1592 children with short stature, whose height, weight, body mass index (BMI), GH peak in two GHSTs, IGF-1 concentration and bone age (BA) were assessed. The cut-off of GH peak in two GHSTs between GHD and idiopathic short stature (ISS) was 10.0 µg/L; additionally, a lower cut-off of 7.0 µg/L was used in repeated analysis. Univariate statistical analyses and classification models were used to identify variables related to the normal and subnormal results of GHST. **Results**: Depending on the cut-off of GH peak (10.0 vs. 7.0 µg/L), GHD was diagnosed in 604 vs. 279 patients (37.9% vs. 17.5%). Children with GHD had significantly lower (*p* < 0.001) BMI SDS and IGF-1 SDS than ones with ISS for both cut-offs of GH peak. Overnutrition was associated with the lowest GH peak but the highest IGF-1 SDS; the opposite results were observed in undernutrition. A decision tree predicted GHD in 156 patients, in 149 based on BMI SDS > 0.91. A Naïve Bayes classifier predicted GHD in 118 cases, with BMI SDS and IGF-1 SDS being the only significant variables. The best multilayer perceptron (MLP) neural network predicted GHD in 310 patients, while a logistic regression model did so in 269 patients. **Conclusions**: Interpretation of GHST should include the patient’s nutritional status in order to avoid overdiagnosis of GHD in overweight and obese children.

## 1. Introduction

### 1.1. Background

Despite 40 years of availability of recombinant human growth hormone (GH) for replacement therapy, the diagnosis of isolated GH deficiency (GHD) remains a challenge for pediatric endocrinologists. According to the guidelines of Grimberg et al. [1], GHD should be confirmed by a decreased GH peak in stimulation tests (GHST), though with a strong recommendation against using it as a sole diagnostic criterion. The Growth Hormone Research Society [2] presented the statement that the diagnosis of GHD should be based on auxologic data, pituitary magnetic resonance imaging (MRI) and laboratory tests, including measurement of insulin-like growth factor-1 (IGF-1) and insulin-like growth factor binding protein-3 (IGFBP-3), followed by GHST. In the classification of pediatric endocrine diagnoses of the European Society for Paediatric Endocrinology (ESPE) [3], GHD is a synonym of IGF-1 deficiency. According to Wit et al. [4], the likelihood of GHD should be assessed before qualifying children to GHST, with measurement of IGF-1 concentration being an important part of laboratory screening. There are also reports indicating a high rate of false-positive GHST results [5,6,7].

The relationships between nutritional status and the results of GHST, with blunted GH response in overweight or obese subjects, have been well documented [1,8,9,10,11]; the same was confirmed in our recent study [12]. Interestingly, the first report of Loche et al. [13], showing reduced GH response to stimulation in obese children and linking this effect to IGF-1 (then referred to as somatomedin-C)-mediated inhibition, was published in 1987. In 2021, Abawi et al. [14] published a systematic review and meta-analysis concerning the impact of body mass index (BMI) on the results of GHST in the pediatric population and proposed using cut-off values of GH peak adjusted for weight status.

### 1.2. Knowledge Gap

The diagnostic controversy and the risk of overdiagnosing GHD mainly concern patients with an idiopathic, isolated deficit of GH, given that in those with anatomic or genetic defects related to impaired GH secretion and/or multiple pituitary hormone deficiency, the diagnosis does not seem doubtful [1,6]. In a recent paper, Kamoun et al. [8] concluded that the problem of the poor sensitivity and specificity of GHST had not been resolved since the 1960s and undoubtedly led to misdiagnosis and likely to overtreatment of patients fulfilling the established diagnostic criteria of GHD. There is also no worldwide consensus on an approach to interpreting the results of GHTS with respect to the nutritional status of children.

The need for assessment of the pre-GHST clinical probability of GHD may be addressed with the use of advanced computational methods of machine learning to create models to predict GHD, based on a patient’s history, auxological data, and the assessment of bone age (BA) and IGF-1 concentrations.

### 1.3. Study Aims

The aim of this study was to assess the relationships between the nutritional status of patients and the results of hormonal tests assessing the somatotropic axis (GH peak in GHST and IGF-1 secretion) and to create prediction models of GHD (defined by subnormal results of GHST) in children with short stature, depending on selected auxological parameters and IGF-1.

## 2. Materials and Methods

### 2.1. Study Design and Patient Population

This retrospective, noninterventional, single-center case–control study included 1592 children (985 boys, 607 girls), age 10.3 ± 3.4 years (mean ± SD), with short stature, defined by patient’s height below 3rd centile for age and sex, according to the national standards [15], diagnosed in a single reference center of pediatric endocrinology in Poland; the same group was a subject of our previous study [12]. Medical records from 2887 hospitalizations of children, referred to our center due to short stature between 2003 and 2020, were screened for eligibility. The medical history of each child was reviewed, and we excluded the patients with the diseases that might affect growth rate and/or GH secretion: congenital malformations, genetic defects, acquired GHD, multiple pituitary hormone deficiency and chronic diseases (for details, see Figure S1 in Supplementary Materials). All the patients were of the same ethnicity (Caucasian race, Polish nationality). Due to the significant rate of missing data on the perinatal period (gestational age, birth weight and length), parental heights, pubertal stage at diagnosis and/or height velocity of the patients (especially in children who were lost to follow-up), and socioeconomic status of the families, these potentially important variables could not be included in the analysis.

The manuscript was prepared according to the FloSTROBE Statement for case-control studies (see File S1 in Supplementary Materials). Minimal dataset is available in Supplementary Materials (File S2).

### 2.2. Clinical and Laboratory Data Collection

The routine diagnostic evaluation, performed for each child, included the following assessments:

- Height and weight were measured, and height standard deviation score (hSDS) was calculated for age and sex according to reference charts of Palczewska and Niedźwiecka [15], BMI and BMI SDS for age and sex calculated with LMS (Lambda–Mu–Sigma) method, according to the reference data of Kułaga et al. [16,17].
- Two separate GHSTs were performed on consecutive days using pharmacological stimulation with clonidine (0.15 mg/m^2^, orally, GH concentrations measured every 30 min from 0 to 120 min of the test) and glucagon (0.03 mg/kg, not exceeding 1.0 mg, intramuscularly). GH concentrations were measured at 0, 90, 120, 150 and 180 min of the test.
- IGF-1 concentration was measured and its SDS calculated (IGF-1 SDS) for age and sex, according to the reference data of Elmlinger et al. [18], using the formula for log-transformed data provided by Blum and Schweitzer [19].
- Bone age (BA) was assessed according to the Greulich–Pyle standards [20], using X-ray images of non-dominant (in small children, left) hand and wrist, performed simultaneously with GHST or up to 6 months before the hormonal diagnostics (this approach was related to the requirements of radiological protection and the time intervals between subsequent standards of BA in the used atlas). BA-to-chronological age (CA) ratio was also calculated as a measure of BA delay.

According to the diagnostic standards, all the patients completed all the tests listed above; there were no patients with any of these data missing.

According to the national rules for the reimbursement of GH therapy, the cut-off value of GH peak in two GHSTs for the diagnosis of GHD (years 2003–2020) and data collection was 10.0 µg/L (in Poland, this threshold has not been changed up to now). The detailed national rules are published quarterly in Announcements of the Polish Minister of Health (the last version is available at https://www.gov.pl/web/zdrowie, accessed on 15 February 2026). Children with GH peak over 10.0 µg/L were diagnosed with idiopathic short stature (ISS). It seems important to stress that, in line with the same recommendations, there was no requirement to document IGF-1 deficiency to establish a diagnosis of GHD.

Considering that the lower cut-offs for GH peak in GHST are currently suggested, we decided to repeat the most important parts of the analysis with the threshold of GH peak for GHD and ISS decreased to 7.0 µg/L. The relevant results are available in the Supplementary Materials. In the context of present study, GHD and ISS should be considered as the labels of the groups with respect to the results of GHST.

Concentrations of GH were measured using the two-site chemiluminescent enzyme immunometric assay (hGH IMMULITE, DPC, Los Angeles, CA, USA) for the quantitative measurement of human GH, calibrated to the WHO IRP 80/505 standard or to 98/574 standards in different years. Even though different laboratory kits were used over 18 years, there was no recommendation to adjust the threshold value of GH peak with respect to the used laboratory method of determining GH concentrations [1,2].

Concentrations of IGF-1 were measured with the solid-phase, enzyme-labelled chemiluminescent immunometric assays (IMMULITE, DPC), calibrated to WHO NIBSC 1st IRR 87/518 and, then, since 2017, with the new IGF-1 assays, standardized to the WHO 1st International Standard 02/254, with appropriate conversion of the results, according to the equation provided in the Siemens Healthcare in Diagnostics Customer Bulletin for IMMULITE^®^ 2000 Immunoassay System [21].

### 2.3. Statistical Analysis

Normality of data distribution was assessed using the Shapiro–Wilk test and was not met for most variables. Thus, the descriptive statistics were presented as medians and interquartile ranges (Q1:Q3). The analysis included both the raw data and the variables based on the same parameters normalized and transformed to the SDS values for age and sex. 

Appropriate non-parametric tests for independent variables were used in the statistical analyses—the Mann–Whitney U test for comparisons between two groups, while the Kruskal–Wallis test with post hoc comparisons for more than two groups. 

For the comparisons of frequencies, the chi-square test was used.

Classification models were constructed using the following input (independent) variables: patient’s age (CA), sex, BA delay (expressed as BA/CA ratio), height SDS, BMI SDS and IGF-1 SDS for age and sex. The output (dependent) qualitative variable was the diagnosis (GHD or ISS), i.e., GH peak in two GHSTs below or over 10.0 µg/L. Several methods for creating models were applied:

- Logistic regression: A standard tool used to model dichotomous (binary) outcomes; all six independent variables were used for the model development with the forward stepwise variable selection, and the significance of variables was assumed at *p* < 0.05. Odds ratios were examined to assess the direction and strength of the dependence between inputs and output.
- Decision tree: A tool that uses a tree-like model for dividing a statistical sample into classes of observations with similar properties; in this technique, the estimated prior probabilities of particular outputs (in our study, of GHD and ISS) are also included. We analyzed the decision rules and cut-off of continuous variables to provide the interpretation of the effects of particular predictors.
- Naïve Bayes classifier: Another method used to solve the problem of sorting decision classes, which takes into account the conditional probability of occurrence of a given feature.
- Artificial neural networks (ANNs): Modeling techniques that do not require any prior assumptions about the nature of the functions connecting independent and dependent variables and that have the ability to automatically eliminate irrelevant (redundant) variables from the model and minimize the error.

All statistical analyses were performed with Statistica 13.1 (TIBCO Software Inc., Palo Alto, CA, USA).

## 3. Results

The diagnosis of GHD based on a GH peak below 10.0 µg/L in the two GHSTs was established in 37.9% of patients, with similar frequency in boys (38.4%) and girls (37.2%); the remaining ones were labelled as ISS. Decreasing the GH peak cut-off from 10.0 µg/L to 7.0 µg/L reduced the number of patients meeting the assumed criteria of GHD from 604 to 279 cases.

### 3.1. Basic Statistics and Univariate Analysis

The characteristics of the study group and comparison of the subgroups diagnosed with GHD and ISS (according to GH peak in two GHST with the cut-off equal to 10.0 µg/L) are presented in Table 1 (for characteristics of the subgroups GHD < 7 and ISS ≥ 7, divided according to the cut-off of 7.0 µg/L, see Table S1 in the Supplementary Materials).

As GHD should be considered as a cause of secondary IGF-1 deficiency, the first part of the analysis was devoted to the assessment of relationships between GH peak and IGF-1 SDS.

After dividing the patients with respect to the diagnosis, IGF-1 SDS was significantly lower (*p* < 0.001) in GHD than in the ISS group (for cut-off of 10.0 µg/L, see Table 1; for cut-off of 7.0 µg/L, see Table S1 in the Supplementary Materials), however with a significant but only weak correlation between the GH peak and IGF-1 SDS (r = 0.223, *p* < 0.05).

**Figure 1:**
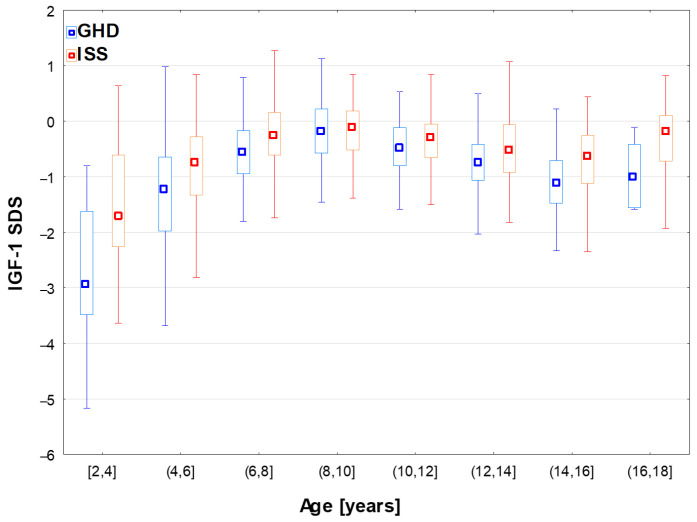
Age-related fluctuations in IGF-1 SDS in the patients diagnosed with GHD and ISS; the results are presented as median (point), Q1–Q3 (box) and non-outlier range (whiskers).

Median values of IGF-1 SDS in the whole GHD group and in the subgroups aged 6–14 years were over −1.0 (see Table 1 and Figure 1, respectively). Somewhat surprisingly, the differences in IGF-1 SDS across age groups were even more pronounced than those between GHD and ISS groups (see Figure 1).

Significant sex-related differences were observed in the whole study group for the majority of the analyzed variables, except GH peak, BMI and BMI SDS (see Table 2). Most of these differences remained significant after dividing the patients with respect to the diagnosis, and the differences in BMI SDS remained insignificant for both GHD and ISS (detailed data for boys and girls in the subgroups with GHD and ISS are presented in Tables S2 and S3 in the Supplementary Materials).

**Figure 2:**
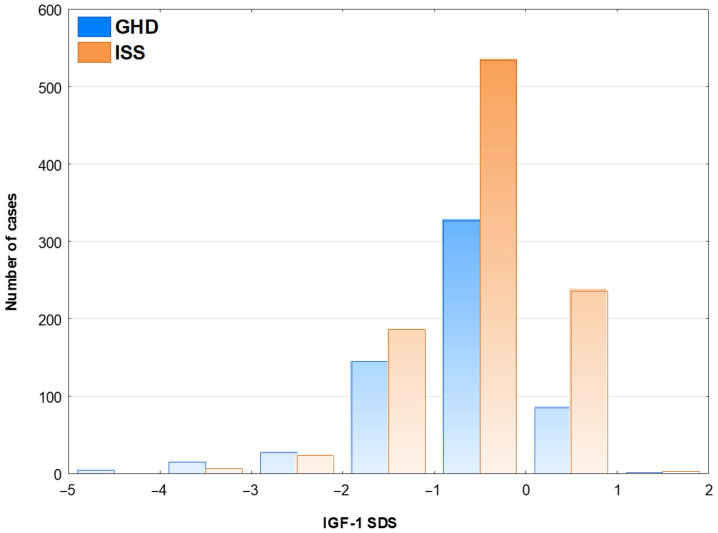
Number of patients with GHD and ISS with respect to IGF-1SDS.

The frequency of GHD (defined by GH peak in two GHST below 10.0 µg/L) exceeded 50% only in patients with IGF-1 SDS below −2.0 (see Figure 2). Moreover, 23.9% of boys and 28.5% of girls with IGF-1 SDS over 0.0 fulfilled the defined diagnostic criteria of GHD. After decreasing the cut-off of GH peak to 7.0 µg/L, the frequency of GHD exceeded 50% only in patients with IGF-1 SDS below −3.0 (see Figure S2). This change decreased the frequency of GHD among children with IGF-1 SDS over 0.0 to only 11.9% in boys and 9.2% in girls.

The patients were subsequently stratified according to their nutritional status into the following groups:

- Norm: normal weight, BMI SDS from −2.0 to 1.0;
- Under: underweight, BMI SDS below −2.0;
- Over: overweight or obese, BMI SDS over 1.0.

Patients with obesity, i.e., those with BMI SDS over 2.0, were included in the Over group, due to a relatively small number of such cases (four boys and three girls with GHD, and two boys with ISS). The only significant differences between the obese and overweight patients were those in BMI and BMI SDS (see Table 3); however, a small group size with obesity should be considered to properly interpret the insignificant results.

The incidence of undernutrition was 6.5% in the GHD group and 10.9% in the ISS group. Conversely, the incidence of overnutrition (overweight or obesity) was 13.4% in the GHD group and only 4.4% in the ISS group (for detailed data, see Table 4). Decreasing the cut-off of the GH peak to 7.0 µg/L increased the incidence of overnutrition to 20.8% in GHD < 7 and 5.1% in the ISS ≥ 7 group (for details, see Table S4 in the Supplementary Materials).

The frequencies of GHD and ISS differed significantly between the groups with different nutritional status in the whole cohort, in boys (*p* < 0.001) and in girls (*p* = 0.0378); similar results were obtained for GHD < 7 and ISS ≥ 7 (all differences significant, *p* < 0.001).

**Figure 3:**
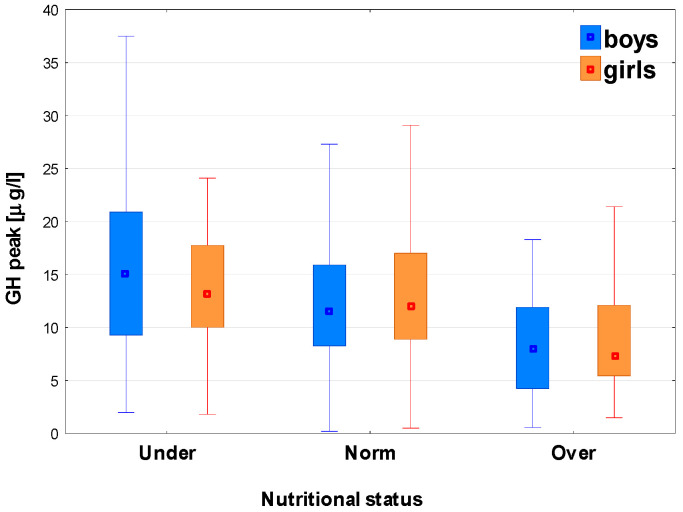
The relationships between the nutritional status and GH peak in two GHSTs in boys and girls; the results are presented as median (point), Q1–Q3 (box) and non-outlier range (whiskers).

**Figure 4:**
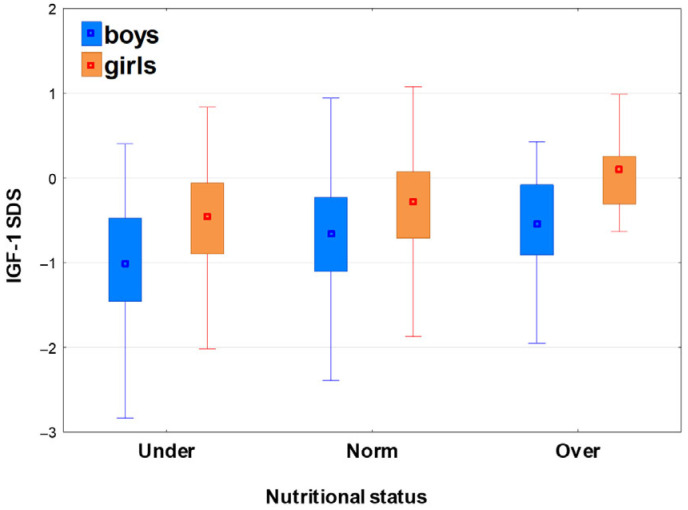
The relationships between the nutritional status and IGF-1 concentrations in boys and girls; the results are presented as median (point), Q1–Q3 (box) and non-outlier range (whiskers).

After classifying the patients with respect to nutritional status, significant differences (*p* < 0.001) were observed in GH peaks for the whole group, as well as for boys and girls; in post hoc analysis, all the differences between the particular subgroups were significant, except between the groups Norm and Under in girls (see Table 3 and Figure 3). Significant differences (*p* < 0.001) were also observed in IGF-1 SDS among the groups with different nutritional status for the whole group, as well as for boys and girls; in the post hoc analysis, all the differences between particular subgroups were also significant, except between Norm and Over in boys and between Norm and Under in girls (see Table 5 and Figure 3 and Figure 4).

It should be stressed that the median GH peak was lower, while the median IGF-1 SDS was higher in the Over than in the Norm group, whereas the Under group had the highest median GH peak together with the lowest median IGF-1 SDS (see Table 5 and Figure 3 and Figure 4).

After dividing the patients with respect to the diagnosis, we observed similar relationships between the IGF-1 concentrations and the nutritional status, i.e., higher values of the median IGF-1 SDS in groups with higher BMI SDS. Patients with GHD had lower IGF-1 SDS than ones with ISS in each category of nutritional status. In post hoc analysis, all the differences in IGF-1 SDS were significant, except for those between Norm and Under in GHD and between Norm and Over in ISS. Notably, the median IGF-1 SDS was higher in patients with GHD and the nutritional status Over than in ones with ISS and Norm or Under (see Table 6) (the relationships between the nutritional status and IGF-1 SDS in the GHD < 7 and ISS ≥ 7 groups were similar; see Table S5 in the Supplementary Materials).

### 3.2. Classification Models

The second part of the analysis focused on developing classification models enabling the prediction of a subnormal (GHD) or normal (ISS) GH peak in two GHSTs with the use of machine learning techniques.

The logistic regression model with a forward stepwise variable selection classified 269 patients to the GHD group (including 167 with diagnosed GHD and 102 with ISS) and the remaining 1323 (including 886 diagnosed with ISS and 437 with GHD) to the ISS group, keeping only BMI SDS and IGF-1 SDS as the significant variables (*p* < 0.001) with odds ratios 1.55 (95% CI: 1.40–1.72) for BMI SDS and 0.54 (95% CI: 0.46–0.62) for IGF-1 SDS.

**Figure 5:**
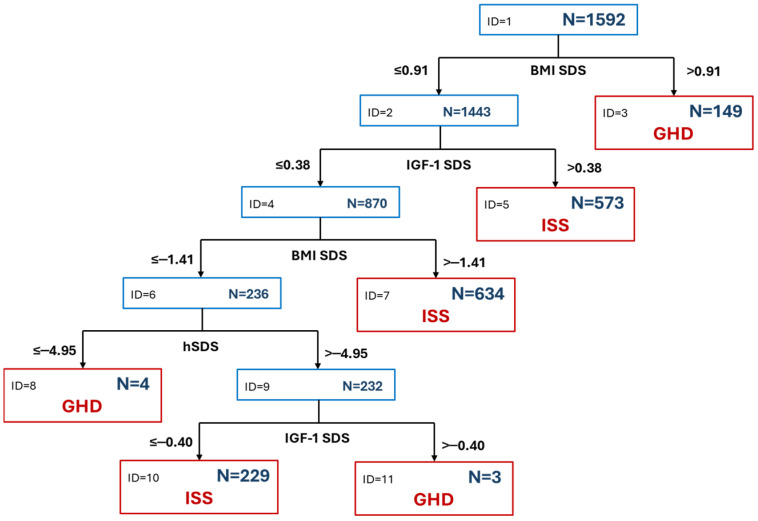
Classification tree based on the auxological indices and IGF-1 SDS of the patients. Terminal Nodes (Leaves): red boxes. Root Node (ID = 1) and Decision Nodes: blue boxes. N: number of cases.

The classification tree created a model, in which BMI SDS > 0.91 was predictive for GHD, while among the remaining 1443 patients with BMI SDS ≤ 0.91, only 7 were classified as the GHD group. Consequently, BMI SDS with a cut-off at the level of 0.91 emerged as the only clinically relevant predictor of GHD (see Figure 5). Even though 604 patients had a decreased GH peak in GHST, the obtained model classified only 156 patients to the GHD group. Nevertheless, this model correctly classified around 65% of the patients, including 938 out of 988 (94.8%) of individuals diagnosed with ISS but only 106 out of 498 (17.5%) children diagnosed with GHD (qualifying most of them as ISS).

The Naïve Bayes classifier predicted GHD in only 118 patients (7.4%), including 74 diagnosed with GHD and 44 with ISS. The only variables that differentiated between GHD and ISS in this model were BMI SDS and IGF-1 SDS. The overall classification accuracy of the Naïve Bayes model was approximately 65%.

Multilayer perceptron (MLP) neural networks correctly classified over 70% of patients. The characteristics of the MLP network with the best accuracy are presented in Table 7. This model correctly classified 310 (51.3%) of the patients diagnosed with GHD, while the remaining 294 (48.7%) children with decreased GH peak in GHST were classified as ISS. Conversely, 855 (86.5%) of children diagnosed with ISS were classified correctly, with only 133 (13.5%) classified as GHD.

The summary of the models for the diagnoses of GHD and ISS (cut-off of GH peak 10 µg/L) is presented in Table 8. Further validation of the models has been omitted due to their low performance in the training set, which excluded the possibility of their implementation.

## 4. Discussion

The results of our study confirmed known associations between nutritional status and GH secretion [1,11], as children with overnutrition had lower GH peaks in GHST than those with normal weight or underweight. The median GH peak in children with overnutrition was below 10.0 µg/dL, which meant a diagnosis of GHD in the majority of them. Such a high frequency of GHD appears unlikely, particularly given a significant proportion of normal IGF-1 concentrations in this group.

The negative association between BMI SDS and the GH peak in GHST has also been confirmed in a large group of patients by Thieme at al. [9]. Abawi et al. [14] published a systematic review and meta-analysis devoted to the impact of BMI on the results of GHST in children and adolescents, including the data of 5135 children from 58 studies. The authors calculated that a one-point increase in BMI SDS decreases the GH peak by 11.6%. Based on their meta-analysis, the authors proposed weight-adjusted (lower) cut-offs for the GH peak in GHST for children with overweight and obesity.

Although GHD was defined as the secondary IGF-1 deficiency in “The ESPE Classification of Paediatric Endocrine Disorders” over 20 years ago [3], the significance of IGF-1 assessment and the interpretation of this test in diagnosing GHD are still a matter of discussion. In the previously quoted Guidelines from 2016 [1], measurement of IGF-1 concentrations has been recommended but mainly as a tool for monitoring the adherence to the treatment and for lowering recombinant human GH (rhGH) doses that are too high. In 2022, Wit et al. [4] measured IGF-1 during a laboratory screening for GHD, interpreting the obtained results with respect to the pubertal stage. The meta-analysis of the diagnostic value of IGF-1 in GHD showed that IGF-1 SDS below −2.0 had quite good sensitivity and specificity for the diagnosis based on the results of GHST [22]. In line with these observations, in our study, the frequency of GHD exceeded 50% only in patients who had IGF-1 SDS below −2.0.

Previous studies examining the relationships between GH and IGF-1 secretion and nutritional status yielded inconsistent results. In the study of Stanley et al. [10], BMI SDS correlated negatively with the GH peak, while there was no association between GH peak and IGF-1. According to the review of Savastano et al. [23], obesity led to a reduced GH response in GHST that might result in reduced IGF-1 levels.

In a study including 9400 serum samples, Hörenz et al. [24] stated that in obese children, IGF-1 levels were higher, while in obese adults, they were lower than in appropriate reference groups with a normal nutritional status. On the contrary, very recently, Negrea et al. [25] reported that IGF-1 concentrations depended on age rather than on the weight status of children.

In our cohort, children with overnutrition had the lowest median GH peak concomitant with the highest values of IGF-1 SDS values, while, conversely, underweight children had the highest median GH peak and the lowest IGF-1 SDS. The median value of IGF-1 SDS was even slightly higher in the overweight children diagnosed with GHD than in non-overweight children diagnosed with ISS. This finding clearly confirms the low reliability of diagnosing GHD solely on the basis of the GHST results. In this context, it is noteworthy that weight loss in children with obesity was associated with an increase in GH secretion but not unequivocally with an increase in IGF-1 [26].

The postulated explanation of these findings might be the promoting effect of hepatic insulin on GH sensitivity (by controlling the expression of GH receptors) and, through this, on liver IGF-1 production [27]; however, it was not a subject of our research. Additional support for this statement could be a better growth response to rhGH therapy in children with higher BMI SDS [28,29]. There are also reports indicating that ghrelin and sirtuin may be involved in linking BMI with GH and IGF-1 [30,31]. Issues related to the effect of childhood obesity on growth and on the results of GHST have been discussed in a recent paper by Karachliou et al. [32].

It seems important that, according to current knowledge, the diagnosis of GHD should also be considered unlikely in children with IGF-1 SDS over 0.0 [1,4]. Nevertheless, in our study, every fourth of the patients with IGF-1 SDS over 0.0 had a decreased GH peak in the two GHSTs, thus meeting the assumed criteria for GHD. These results indicate that skipping the IGF-1 concentration and the nutritional status of children while diagnosing GHD may lead to an unreliable diagnosis. There is a need to modify the diagnostic criteria by taking into account these variables in order to avoid overdiagnosing GHD.

It should be recalled here that the cut-off value of the GH peak in two GHSTs for the diagnosis of GHD was established at the level of 10.0 µg/L, according to national rules. Lowering the cut-off of the GH peak to 7.0 µg/L reduced the number of patients fulfilling the established criteria of GHD by more than 50%, as well as the proportion of patients with IGF-1 SDS over 0.0 having subnormal GH peaks in GHST. Undoubtedly, using the lower cut-offs for the interpretation of GHST, as proposed in current guidelines [1,2,33], should decrease the number of children diagnosed with GHD and subjected to rhGH therapy. Nevertheless, the problems related to the opposite relationships of the GH peak and IGF-1 SDS with nutritional status have remained unresolved. Unfortunately, there is no clear recommendation to use different GH peak cut-offs in GHST with respect to the nutritional status of the patients; in Poland, there is also no requirement to confirm the IGF-1 deficiency for the diagnosis of GHD.

In this context, a recent study by Ozdemir Dilek and Özgüç Comlek [34] seems very interesting. They proposed a two-step diagnostic algorithm including GHST followed by measurement of IGF-1 and the observation of growth velocity to reduce overdiagnosing GHD in overweight/obese children.

In our study, some differences in the analyzed variables between boys and girls were observed. The importance of the gender-related differences concerning the effect of BMI on the results of GHST was recently stressed by Ari et al. [35]. Interestingly, these differences were observed in prepubertal children. It seems possible that a patient’s gender should be one of the variables taken into account when interpreting GHST.

Machine learning techniques are constantly gaining popularity and are used successfully to create complex models, not only in medicine. In a recent study by Zhu et al. [36], different methods, including decision trees, random forest, MLP networks and logistic regression, have been used for constructing height prediction models for children treated with rhGH, and the best accuracy was shown for the random forest and MLP models, with patients’ age, BA delay, height SDS and BMI SDS but not IGF-1 as the most influential variables in the MLP model. In our previous study, good accuracy was also documented for MLP models in predicting the growth response to rhGH therapy, with the pre-treatment growth and IGF-1 deficiency as the main predictors [37]. Other studies included additional variables, Sella MRI findings [38] or transcriptomic data [39], together with clinical parameters in the prediction models of GHD and ISS. In the model created by the external gradient boosting algorithm, BMI SDS was listed among the major contributors to prediction, together with the difference between CA and BA, weight SDS and growth velocity; also, the radiomic features added incremental value to the prediction [38]. In the study of Garner al. [39], no significant difference was reported in auxological parameters (height SDS, weight SDS, BMI SDS) and IGF-1 SDS between the GHD and ISS groups, while the random forest algorithm using a combination of gene expression data presented high accuracy. A limitation of that study, important in the context of the lack of statistical significance, may be the very small number of patients (8 in the GHD and 16 in the ISS group). Nevertheless, patients with GHD had higher BMI SDS and lower IGF-1 SDS than those with ISS.

In our study, a relatively simple classification task was set; however, the solutions obtained were not satisfactory. The most important problem of all the created models was classifying the high proportion of patients diagnosed with GHD as ISS. The reasons underlying this observation are not obvious. Classification trees are used to divide a statistical sample into classes of observations with similar properties. In our cohort, BMI SDS showed the only relevant difference between GHD and ISS, and most of the patients diagnosed with GHD (i.e., ones with decreased GH peak in GHST) were, according to the model, more similar to ISS than to GHD. The strength of BMI SDS as a predictor in this model made a real assessment of the prognostic value of other variables impossible. A Naïve Bayes classifier is a relatively simple tool. Although this method should take into account the actual probability of belonging to one of the classes (in our study, to GHD or ISS), it classified the lowest number of patients to the GHD group, with a relatively large number of misclassifications. It must be admitted that this tool might not have been optimal because Naïve Bayes classifiers consider each of the features of the object as independent and all features as contributing equally to the outcome. Nevertheless, in this model, only BMI SDS and IGF-1 SDS turned out to be significant. MLP neural networks correctly classified (i.e., in line with the results of GHST) the largest number of patients, though still with a substantial number of cases re-classified from GHD to ISS. Unfortunately, this method does not allow for easy discovery of the dependencies underlying the classification decisions.

The limitations of this study include the retrospective design, single-center setting, assay variability across the study period, possible ethnicity-related biases (single ethnicity of all patients), and the lack of external validation. The most important problems were the incomplete records of some patients (missing data on gestational age, birth size, parental heights, pubertal stage and growth rate). In a retrospective study, covering a multi-year period, there was no way to complete this information in a reliable manner. This made the inclusion of these potentially significant variables in the analysis impossible. Further prospective studies could overcome these limitations.

## 5. Conclusions

The results of our study clearly support the recommendation that in children with short stature, distinguishing between GHD and ISS based solely on the results of GHST without considering IGF-1 concentration and the nutritional status is a diagnostic procedure of limited reliability.

Documenting the opposite associations of BMI SDS with the GH peak in GHST (negative) and IGF-1 SDS (positive) in children with short stature is a novel finding that seems important for the proper diagnosis of GHD in the case of overnutrition. The use of the same cut-off value of the GH peak for all GHSTs, irrespective of the patient’s nutritional status, should be reconsidered to reduce GDH overdiagnosis in overweight and obese children, especially in the context of no evidence of IGF-1 deficiency in a significant proportion of them. Appropriate modifications to the diagnostic criteria of GHD are important from the clinical point of view to avoid the unnecessary treatment of misdiagnosed children.

The significance of higher BMI SDS for the prediction of GHD, shown in machine learning models, further supports the need to interpret the results of GHST in the context of the nutritional status of children rather than demonstrating the inability of machine learning techniques to create prediction models.

## Figures and Tables

**Table 1:** Comparison of the patients with GHD and ISS (the cut-off of GH peak 10.0 µg/L).

	All	GHD	ISS	*p* **
Number of cases (boys/girls)	1592 (985/607)	604 (378/226)	988 (607/381)	
CA [years] *	10.9 (7.4; 13.0)	11.1 (7.6; 11.2)	10.7 (7.3; 13.1)	0.85
Height [cm]	128.6 (112.1; 140.5)	129.6 (112.8; 140.0)	127.2 (111.8; 141.0)	0.78
hSDS for age and sex	−2.47 (−2.91; −2.17)	−2.43 (−2.95; −2.15)	−2.49 (−2.90; −2.19)	0.72
Body mass [kg]	26.1 (18.5; 34.7)	28.0 (19.2; 36.0)	25.0 (18.3; 35.6)	<0.001
BMI [kg/m^2^]	15.8 (14.6; 17.7)	16.3 (14.9; 18.9)	15.5 (14.4; 17.3)	<0.001
BMI SDS for age and sex	−0.58 (−1.32; 0.07)	−0.39 (−1.10; 0.45)	−0.68 (−1.45; −0.11)	<0.001
GH peak in two GHST [µg/L]	11.7 (8.2; 16.5)	7.3 (5.5; 8.7)	14.9 (12.1; 19.8)	<0.001
IGF-1 [µg/L]	147.0 (89.1; 217.5)	134.0 (81.1; 195.0)	159.0 (95.4; 233.6)	<0.001
IGF-1 SDS for age and sex	−0.52 (−1.01; −0.09)	−0.64 (−1.16; −0.26)	−0.42 (−0.90; −0.02)	<0.001
BA [years]	9.0 (5.5; 11.0)	9.0 (5.5; 11.0)	8.8 (5.0; 11.5)	0.61
BA/CA ratio	0.81 (0.70; 0.90)	0.81 (0.70; 0.89)	0.82 (0.70; 0.91)	0.21

* Data presented as median (Q1; Q3); ** *p*—differences between GHD and ISS in Mann-Whitney U test. Abbreviations: CA—chronological age, hSDS—height standard deviation score, BMI—body mass index, BMI SDS—body mass index standard deviation score, GH—growth hormone, GHST—growth hormone stimulation test, IGF-1—insulin-like growth factor-1, IGF-1 SDS—insulin-like growth factor-1 standard deviation score, BA—bone age.

**Table 2:** Comparison of boys and girls in the whole study group.

	Boys	Girls	*p* **
Number of cases	985	607	
CA [years] *	11.5 (7.3; 13.6)	10.1 (7.5; 12.1)	<0.001
Height [cm]	128.2 (112.6; 142.9)	124.8 (111.5; 136.2)	<0.001
hSDS for age and sex	−2.42 (−2.85; −2.16)	−2.52 (3.00; −2.20)	<0.001
Body mass [kg]	27.5 (19.2; 36.0)	24.5 (18.0; 31.2)	<0.001
BMI [kg/m^2^]	16.0 (14.8; 17.9)	15.5 (14.1; 17.4)	<0.001
BMI SDS for age and sex	−0.57 (−1.26; 0.04)	−0.60 (−1.38; 0.16)	0.58
GH peak in two GHST [µg/L]	11.6 (8.1; 15.9)	11.9 (8.6; 16.9)	0.15
IGF-1 [µg/L]	141.0 (86.0; 206.0)	161.0 (96.6; 236.0)	<0.01
IGF-1 SDS for age and sex	−0.66 (−1.13; −0.24)	−0.28 (−0.68; 0.10)	<0.001
BA [years]	9.0 (5.0; 11.5)	9.75 (5.5; 11.0)	<0.01
BA/CA ratio	0.80 (0.69; 0.88)	0.84 (0.73; 0.92)	<0.001

* Data presented as median (Q1; Q3); ** *p*—differences between GHD and ISS in Mann–Whitney U test. Abbreviations: see Table 1.

**Table 3:** Comparison of patients with obesity and overweight.

	Obesity	Overweight	*p* **
Number of cases (boys/girls)	9 (6/3)	116 (65/51)	
CA [years] *	11.8 (8.4; 13.2)	11.6 (8.8; 13.4)	0.96
Height [cm]	132.6 (121.2; 140.0)	132.2 (119.0; 143.1)	0.91
hSDS for age and sex	−2.27 (−2.41; −2.01)	−2.33 (−2.88; −2.08)	0.30
Body mass [kg]	44.0 (33.6; 58.0)	40.0 (29.7; 50.0)	0.56
BMI [kg/m^2^]	27.0 (23.2; 29.7)	22.6 (20.7; 24.3)	0.047
BMI SDS for age and sex	2.28 (2.08; 2.72)	1.31 (1.16; 1.55)	<0.001
GH peak in two GHST [µg/L]	6.1 (3.4; 8.1)	7.6 (4.7; 12.1)	0.39
IGF-1 [µg/L]	233.0 (177.0; 250.9)	194.8 (126.5; 267.5)	0.86
IGF-1 SDS for age and sex	−0.08 (−0.65; −0.04)	−0.29 (−0.65; 0.14)	0.70
BA [years]	11.0 (4.5; 13.0)	10.25 (7.0; 12.0)	0.75
BA/CA ratio	0.88 (0.85; 0.99)	0.82 (0.70; 0.90)	0.80

* Data presented as median (Q1; Q3); ** *p*—differences between GHD and ISS in Mann–Whitney U test. Abbreviations: see Table 1.

**Table 4:** Number of patients in particular groups depending on the diagnosis and nutritional status.

	Study Group			GHD			ISS		
	All	Boys	Girls	All	Boys	Girls	All	Boys	Girls
All	1592	985	607	604	378	226	988	607	381
Under	147	87	60	39	24	15	108	63	45
Norm	1320	827	493	484	309	175	836	518	318
Over	125	71	54	81	45	36	44	26	18

**Table 5:** GH peak and IGF-1 SDS in relation to the nutritional status of the patients.

	All	Under	Norm	Over	*p* **
GH peak [µg/L] *	13.1 (8.2; 16.5)	13.8 (9.6; 19.5)	11.7 (8.5; 16.6)	7.5 (4.7; 12.0)	<0.001
IGF-1 SDS	−0.52 (−1.01; −0.09)	−0.79 (−1.27; −0.25)	−0.61 (−1.01; −0.11)	−0.41 (−0.65; 0.13)	<0.001

* Data presented as median (Q1; Q3); ** *p*-value—differences between Groups in Kruskal–Wallis test. Abbreviations: GH—growth hormone, IGF-1 SDS—insulin-like growth factor-1 standard deviation score.

**Table 6:** IGF-1 SDS with respect to the diagnosis and nutritional status of the patients.

Group	All	Under	Norm	Over	*p* **
GHD *	−1.04 (−1.55: −0.61)	−1.04 (−1.55: −0.61)	−0.68 (−1.16; −0.29)	−0.35 (−0.74; 0.08)	<0.001
ISS	−0.42 (−0.90; −0.02)	−0.56 (−1.14; −0.19)	−0.42 (−0.89; −0.02)	−0.16 (−0.54; 0.18)	0.005

* Data presented as median (Q1; Q3); ** *p*-value—differences between Groups in Kruskal–Wallis test. Abbreviations: IGF-1 SDS—insulin-like growth factor-1 standard deviation score, GHD—growth hormone deficiency, ISS—idiopathic short stature.

**Table 7:** Characteristics of the best MLP neural network.

Network	Training Accuracy	Training Algorithm	Error Function	Hidden Activation	Output Activation
MLP 7-11-2	73.2	BFGS 157	Cross entropy	Logistic	Softmax

Abbreviations: MLP—multilayer perceptron.

**Table 8:** Summary of the classification models.

	True Positive	False Negative	False Positive	True Negative	Accuracy (%)	Sensitivity (%)	Specificity (%)
Logistic regression	167	437	102	886	66.2	27.6	89.7
Decision tree	106	498	50	938	65.6	17.5	94.9
Naïve Bayes	74	530	44	944	63.9	12.3	95.5
MLP	310	294	133	855	73.2	48.7	86.5

## Data Availability

The data supporting the findings of this study are included in the Supplementary Materials. Further inquiries can be directed to the corresponding author.

## Supplementary Materials

The following supporting information can be downloaded at: https://www.mdpi.com/article/10.3390/jcm15093333/s1, Figure S1. Flowchart of patient selection. Figure S2. Number of patients with GHD < 7 and ISS ≥ 7 with respect to IGF-1SDS. Table S1. Comparison of the patients with GHD < 7 and ISS ≥ 7 (cut-off of GH peak in GHST 7.0 µg/L). Table S2. Comparison of boys and girls with GHD (cut-off of GH peak 10.0 µg/L). Table S3. Comparison of boys and girls with ISS (cut-off of GH peak 10.0 µg/L). Table S4. Number of patients in particular groups depending on the diagnosis and nutritional status (cut-off of GH peak 7.0 µg/L). Table S5. IGF-1 SDS with respect to the diagnosis and nutritional status of the patients (cut-off of GH peak in GHST 7.0 µg/L). File S1. STROBE checklist. File S2. Minimal dataset.

## Abbreviations

The following abbreviations are used in this manuscript:

ANN	Artificial neural network
BA	Bone age
BMI	Body mass index
CA	Chronological age
CI	Confidence interval
ESPE	European Society for Pediatric Endocrinology
GH	Growth hormone
GHD	Growth hormone deficiency
GHST	Growth hormone stimulation tests
hSDS	Height Standard deviation score
IGF-1	Insulin-like growth factor-1
ISS	Idiopathic short stature
MLP	Multilayer perceptron
MRI	Magnetic resonance imaging
Norm	Normal weight
Over	Overweight
rhGH	Recombinant human growth hormone
SD	Standard deviation
SDS	Standard deviation score
Under	Underweight
