# Supplementary material for: Nutritional Status of Children with Short Stature Is Oppositely Associated with Growth Hormone Peak in Stimulation Tests and Insulin-like Growth Factor-1 Concentration
Source: J Clin Med. 2026 Apr 27;15(9):3333. doi: 10.3390/jcm15093333 (PMC13163395; doi:10.3390/jcm15093333)
Supplement: Supplementary file 1 [file jcm-15-03333-s001.zip › jcm-4187021-supplementary/jcm-4187021-STROBE checklist.pdf]

FloSTROBE Statement—Checklist of items that should be included in reports of ***case-control studies***

	Item No	Recommendation	Our study
**Title and abstract**	1	(*a*) Indicate the study's design with a commonly used term in the title or the abstract	Study is defined as case-control in Methods in the abstract
		(*b*) Provide in the abstract an informative and balanced summary of what was done and what was found	See abstract
**Introduction**			
Background/rationale	2	Explain the scientific background and rationale for the investigation being reported	Section 1.1 explains background and section 1.2 rationale for current study
Objectives	3	State specific objectives, including any prespecified hypotheses	Section 1.3 defines study aims
**Methods**			
Study design	4	Present key elements of study design early in the paper	See section 2.1
Setting	5	Describe the setting, locations, and relevant dates, including periods of recruitment, exposure, follow-up, and data collection	Section 2.1 specifies location and dates, section 2.2 collected data
Participants	6	(*a*) Give the eligibility criteria, and the sources and methods of case ascertainment and control selection. Give the rationale for the choice of cases and controls	Eligibility criteria are defined in section 2.1, definitions of compared groups in 2.2
		(*b*) For matched studies, give matching criteria and the number of controls per case	Not applicable, no matching used
Variables	7	Clearly define all outcomes, exposures, predictors, potential confounders, and effect modifiers. Give diagnostic criteria, if applicable	Variables, outcomes and diagnostic criteria are specified in section 2.2
Data sources/ measurement	8*	For each variable of interest, give sources of data and details of methods of assessment (measurement). Describe comparability of assessment methods if there is more than one group	See section 2.2
Bias	9	Describe any efforts to address potential sources of bias	Efforts to address potential sources of bias are described in 2.1 and 4

Study size	10	Explain how the study size was arrived at	See. Section 2.1 and Supplementary materials (Fig. 1S)
Quantitative variables	11	Explain how quantitative variables were handled in the analyses. If applicable, describe which groupings were chosen and why	Section 2.2 describes quantitative clinical variables and derived features (e.g. standard deviation scores)
Statistical methods	12	(*a*) Describe all statistical methods, including those used to control for confounding	See section 2.3
		(*b*) Describe any methods used to examine subgroups and interactions	See Section 2.3
		(*c*) Explain how missing data were addressed	Section 2.1 explains missing data issue
		(*d*) If applicable, explain how matching of cases and controls was addressed	Not applicable
		(*e*) Describe any sensitivity analyses	See Section 2.3
**Results**			
Participants	13*	(a) Report numbers of individuals at each stage of study—eg numbers potentially eligible, examined for eligibility, confirmed eligible, included in the study, completing follow-up, and analysed	See. Section 2.1 and Supplementary materials (Fig. 1S)
		(b) Give reasons for non-participation at each stage	See. Section 2.1 and Supplementary materials (Fig. 1S)
		(c) Consider use of a flow diagram	Flow diagram is in Supplementary materials (Fig. 1S)
Descriptive data	14*	(a) Give characteristics of study participants (eg demographic, clinical, social) and information on exposures and potential confounders	Participants are characterized in section 3.1 and Table 1
		(b) Indicate number of participants with missing data for each variable of interest	See Section 2.2
Outcome data	15*	Report numbers in each exposure category, or summary measures of exposure	See Table 1
Main results	16	(*a*) Give unadjusted estimates and, if applicable, confounder-adjusted estimates and their precision (eg, 95% confidence interval). Make clear which confounders were adjusted for and why they were included	See Tables 3 and 4 for unadjusted comparisons, and logistic regression model in section 3.2 for covariate-adjusted analysis

		(*b*) Report category boundaries when continuous variables were categorized	Category boundaries are specified in section 3.1
		(*c*) If relevant, consider translating estimates of relative risk into absolute risk for a meaningful time period	Not applicable
Other analyses	17	Report other analyses done—eg analyses of subgroups and interactions, and sensitivity analyses	Section 3.2 describes statistical models
**Discussion**			
Key results	18	Summarise key results with reference to study objectives	See paragraphs 7, 9 and 12 in the Discussion
Limitations	19	Discuss limitations of the study, taking into account sources of potential bias or imprecision. Discuss both direction and magnitude of any potential bias	Last paragraph of the Discussion lists limitations
Interpretation	20	Give a cautious overall interpretation of results considering objectives, limitations, multiplicity of analyses, results from similar studies, and other relevant evidence	See Conclusions section
Generalisability	21	Discuss the generalisability (external validity) of the study results	See paragraph 9 in the Discussion
**Other information**			
Funding	22	Give the source of funding and the role of the funders for the present study and, if applicable, for the original study on which the present article is based	See Funding section

*Information given separately for cases and controls.

Note: An Explanation and Elaboration article discusses each checklist item and gives methodological background and published examples of transparent reporting. The STROBE checklist is best used in conjunction with this article (freely available on the Web sites of PLoS Medicine at http://www.plosmedicine.org/, Annals of Internal Medicine at http://www.annals.org/, and Epidemiology at http://www.epidem.com/). Information on the STROBE Initiative is available at http://www.strobe-statement.org.
