# Supplementary material for: Nutritional Status of Children with Short Stature Is Oppositely Associated with Growth Hormone Peak in Stimulation Tests and Insulin-like Growth Factor-1 Concentration
Source: J Clin Med. 2026 Apr 27;15(9):3333. doi: 10.3390/jcm15093333 (PMC13163395; doi:10.3390/jcm15093333)
Supplement: Supplementary file 1 [file jcm-15-03333-s001.zip › jcm-4187021-supplementary/jcm-4187021-supplementary final version.pdf]

Supplementary materials include:

1. Flowchart of patients selection (Fig. S1)
2. Comparisons of boys and girls with respect to the diagnosis (GHD and ISS, cut-off of GH peak 10.0 μg/l) (Tables S1 and S2)
3. Univariate analysis, repeated after dividing the patients using cut-off of GH peak 7.0 μg/l into the groups GHD<7 and ISS≥7 (Tables S3-S5, Fig. S2).

Details of patients selection are presented in Figure S1.

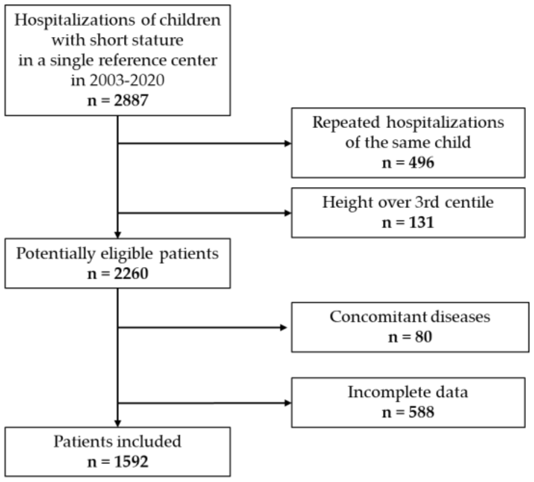

Figure S1. Flowchart of the selection of eligible patients.

Table S1. Comparison of the patients with GHD<7 and ISS≥7 (cut-off of GH peak in GHST 7.0 μg/l).

	All	GHD<7	ISS≥7	p**
Number of cases (boys/girls)	1592 (985/607)	279 (182/97)	1313 (803/510)	
CA [years]*	10.9 (7.4; 13.0)	11.6 (8.0; 13.0)	10.7 (7.3; 13.0)	0.12
Height [cm]	128.6 (112.1; 140.5)	131.8 (113.7; 140.6)	127.4 (111.8; 140.3)	0.15
hSDS for age and sex	-2.47 (-2.91; -2.17)	-2.41 (-2.95; -2.13)	-2.48 (-2.91; -2.18)	0.87
Body mass [kg]	26.1 (18.5; 34.7)	30.0 (20.3; 39.0)	25.5 (18.3; 33.7)	<0.001
BMI [kg/m^2]	15.8 (14.6; 17.7)	17.0 (15.1; 20.3)	15.6 (14.5; 17.4)	<0.001
BMI SDS for age and sex	-0,58 (-1.32; 0.07)	-0.14 (-0.99; 0.91)	-0.66 (-1.37; -0.05)	<0.001
GH peak in two GHST [μg/l]	11.7 (8.2; 16.5)	5.4 (3.9; 6.3)	13.1 (10.0; 17.7)	<0.001
IGF-1 [μg/l]	147.0 (89.1; 217.5)	131.0 (71.2; 190.7)	153.0 (91.9; 222.4)	<0.001
IGF-1 SDS for age and sex	-0.52 (-1.01; -0.09)	-0.76 (-1.31; -0.30)	-0.47 (-0.94; -0.06)	<0.001
BA [years]	9.0 (5.5; 11.0)	9.0 (6.0; 11.0)	8.8 (5.0; 11.0)	0.39
BA/CA ratio	0.81 (0.70; 0.90)	0.82 (0.70; 0.90)	0.81 (0.70; 0.90)	0.66

*Data presented as median (Q1;Q3); **p - differences between GHD and ISS in Mann-Whitney U test.

Abbreviations: CA – chronological age, hSDS – height standard deviation score, BMI – body mass index, BMI SDS – body mass index standard deviation score, GH – growth hormone, GHST – growth hormone stimulation test, IGF-1 – insulin-like growth factor-1, IGF-1 SDS – insulin-like growth factor-1 standard deviation score, BA – bone age.

Table S2. Comparison of boys and girls with GHD (cut-off of GH peak 10.0 μg/l).

	**Boys**	**Girls**	**p****
Number of cases	378	226	
CA [years]*	11.7 (7.3; 13.5)	10.5 (8.2; 11.9)	<0.001
Height [cm]	133.0 (112.0; 141.9)	126.1 (114.0; 135.6)	<0.001
hSDS for age and sex	-2.42 (-2.82; -2.16)	-2.48 (3.07; -2.14)	0.13
Body mass [kg]	29.2 (19,2; 38.0)	27.0 (19.1; 33.7)	<0.001
BMI [kg/m²]	16.3 (15.1; 19.0)	16.3 (14.5; 18.6)	0.23
BMI SDS for age and sex	-0.41 (-1.07; 0.32)	-0.30 (-1.13; 0.62)	0.43
GH peak in two GHST [μg/l]	7.1 (5.4; 8.5)	7.4 (5.9; 9.0)	0.04
IGF-1 [μg/l]	130.5 (72.1; 192.0)	138.6 (88.8; 210.00)	0.09
IGF-1 SDS for age and sex	-0.83 (-1.25; -0.42)	-0.41 (-0.81; -0. 04)	<0.001
BA [years]	9.0 (5.0; 11.5)	8.75 (6.5; 10.0)	0.05
BA/CA ratio	0.80 (0.69; 0.88)	0.83 (0.74; 0.91)	0.003

*Data presented as median (Q1;Q3); **p - differences between GHD and ISS in Mann-Whitney U test. Abbreviations: see Table S1.

Table S3. Comparison of boys and girls with ISS (cut-off of GH peak 10.0 μg/l).

	**Boys**	**Girls**	**p****
Number of cases	607	381	
CA [years]*	11.4 (7.3; 13.8)	10.0 (7.1; 12.3)	<0.001
Height [cm]	130.9 (113.0; 143.5)	123.2 (110.0; 137.0)	<0.001
hSDS for age and sex	-2.43 (-2.87; -2.17)	-2.55 (-2.96; -2.23)	0.002
Body mass [kg]	26.6 (19,2; 35.0)	23.0 (17.5; 30.3)	<0.001
BMI [kg/m²]	15.8 (14.6; 17.5)	15.1 (13.9; 17.0)	<0.001
BMI SDS for age and sex	-0.67 (-1.41; -0.11)	-0.82 (-1.50; -0.10)	0.23
GH peak in two GHST [μg/l]	16.9 (12.0; 19.8)	15.7 (12.3; 19.8)	0.25
IGF-1 [μg/l]	147.0 (91.9; 219.0)	170.0 (102.0; 251.0)	0.01
IGF-1 SDS for age and sex	-0.57 (-1.05; -0.11)	-0.21 (-0.60; 0.15)	<0.001
BA [years]	9.0 (5.0; 12.0)	8.5 (5.5; 11.0)	0.05
BA/CA ratio	0.80 (0.69; 0.89)	0.85 (0.73; 0.93)	<0.001

*Data presented as median (Q1;Q3); **p - differences between GHD and ISS in Mann-Whitney U test. Abbreviations: see Table S1

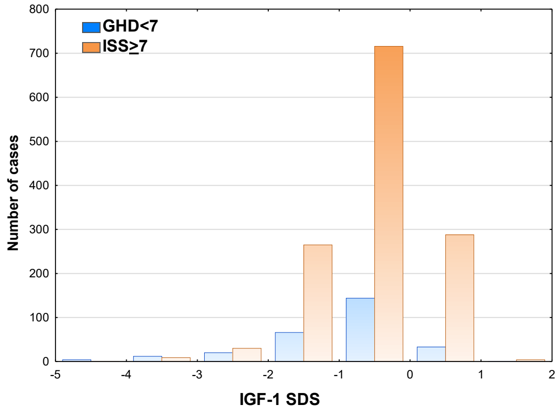

Figure S2. Number of patients with GHD<7 and ISS≥7 with respect to IGF-1SDS.

Table S4. Number of patients in particular groups depending on the diagnosis and nutritional status (cut-off of GH peak in GHST 7.0 μg/l).

	**All**			**GHD<7**			**ISS≥7**		
	**All**	**Boys**	**Girls**	**All**	**Boys**	**Girls**	**All**	**Boys**	**Girls**
All	1592	985	607	279	182	97	1313	803	510
Under	147	87	60	17	12	5	130	75	55
Norm	1320	827	493	204	136	68	1116	691	425
Over	125	71	54	58	34	24	67	37	30

Table S5. IGF-1 SDS with respect to the diagnosis and nutritional status of the patients (cut-off of GH peak in GHST 7.0 μg/l).

**Group**	**All**	**Under**	**Norm**	**Over**	**p****
GHD<7*	-0.76 (-1.31: -0.30)	-1.04 (-1.55: -0.61)	-0.82 (-1.40; -0.39)	-0.49(-0.80; -0.04)	<0.001
ISS≥7	-0.53 (-0.94; -0.06)	-0.62 (-1.23; -0.20)	-0.47 (-0.93; -0.07)	-0.16 (-0.55; 0.28)	<0.001

* Data presented as median (Q1;Q3);

**p-value – differences between Groups in Kruskal-Wallis test

Abbreviations: IGF-1 SDS – insulin-like growth factor-1 standard deviation score, GHD – growth hormone deficiency, ISS – idiopathic short stature
